# PTPN2 Downregulation Is Associated with Albuminuria and Vitamin D Receptor Deficiency in Type 2 Diabetes Mellitus

**DOI:** 10.1155/2018/3984797

**Published:** 2018-08-30

**Authors:** Li Zheng, Wei Zhang, Aimei Li, Yan Liu, Bin Yi, Farid Nakhoul, Hao Zhang

**Affiliations:** ^1^Department of Nephrology, The Third Xiangya Hospital of Central South University, 138 Tongzipo Road, Changsha, Hunan, China; ^2^Diabetic Nephropathy Lab, Baruch Padeh Poriya Medical Center Affiliated to the Faculty of Medicine in Galilee, 15208 Lower Galilee, Israel

## Abstract

**Objective:**

Inflammation plays a major role in albuminuria in type 2 diabetes mellitus (T2DM). Our previous studies have shown that the expression of vitamin D receptor (VDR) is downregulated in T2DM which is closely associated with the severity of albuminuria. In this study, we investigated the expression of anti-inflammatory cytokine protein tyrosine phosphatase nonreceptor type 2 (PTPN2) in T2DM and explored its relationship to albuminuria and VDR.

**Methods:**

101 T2DM patients were divided into three groups based on urinary albumin-to-creatinine ratio (uACR): normal albuminuria (uACR < 30 mg/g, *n* = 29), microalbuminuria (30 mg/g ≤ uACR < 300 mg/g, *n* = 34), and macroalbuminuria (uACR ≥ 300 mg/g, *n* = 38). Thirty healthy individuals were included as controls. Serum was analyzed for PTPN2 and IL-6 expression, and peripheral blood mononuclear cells (PBMCs) were analyzed for PTPN2 and VDR expression. THP-1 cells were incubated with high glucose and further treated with or without paricalcitol, a vitamin D analog. The levels of PTPN2, VDR, IL-6, TNF*α*, and MCP-1 were analyzed. In addition, anti-inflammatory activities of PTPN2 were further explored in THP-1 cells stimulated with high glucose after PTPN2 silencing or overexpression.

**Results:**

PTPN2 expression was downregulated in T2DM with the lowest level observed in macroalbuminuria patients. PTPN2 level positively correlated with VDR but negatively correlated with uACR and IL-6. When stimulated with high glucose, there was an increase in inflammatory factors and a decrease in PTPN2 expression. Treatment with paricalcitol reversed these effects. However, paricalcitol failed to exert anti-inflammatory effects in the setting of PTPN2 knockdown. Thus, low levels of PTPN2 aggravated glucose-stimulated inflammation, while high levels of PTPN2 reduced it.

**Conclusion:**

PTPN2, an anti-inflammatory factor regulated by VDR, was reduced in T2DM CKD stages 1-2. Taken together, our results suggest that therapeutic strategies that enhance PTPN2 may be beneficial for controlling inflammation in T2DM.

## 1. Introduction

Diabetes mellitus (DM) is a prevalent metabolic disease that adversely affects the length and quality of life. Approximately 387 million suffer from DM worldwide [1]. Long-standing diabetes mellitus may finally lead to diabetic kidney disease and even end-stage renal disease [2] and significantly increased mortality [3]. Therefore, there is an urgent need to identify novel therapeutic targets for DM.

The pathogenesis of diabetes mellitus is not entirely clear, but growing evidence has shown that inflammation plays a vital role in the disease development [4–6]. Vitamin D receptor (VDR), a member of the nuclear receptor superfamily, is an important anti-inflammatory mediator that has been studied widely in the pathogenesis of diabetic kidney disease. Patients with diabetes mellitus have varying degrees of vitamin D deficiency [7] which is associated with renal inflammation [8]. Active vitamin D analogs have potent anti-inflammatory properties and have been shown to reduce urine albuminuria *in vivo* [9–11] and *in vitro* [12–14]. The biological effects of vitamin D are mediated by VDR, a ligand-inducible transcription factor that can regulate expression of a gene network [15].

One of the genes closely associated with VDR is PTPN2 [16]. PTPN2, also known as T cell protein tyrosine phosphatase (TCPTP), is an intracellular tyrosine-specific phosphatase that is expressed ubiquitously in epithelial cells, fibroblasts, and endothelial cells and is abundant in hematopoietic and lymphoid cells [17]. PTPN2 has two variants, a 48 kDa form in the endoplasmic reticulum and a 45 kDa form in the nucleus. The nuclear variant translocates to the cytoplasm in response to proinflammatory stimuli. PTPN2 has been implicated in the regulation of insulin signaling and glucose homeostasis [18, 19] and is also associated with chronic inflammatory and autoimmune diseases such as rheumatoid arthritis (RA) [20], Crohn's disease [21], periodontitis [22], and type 1 diabetes mellitus (T1DM) [23]. In humans, PTPN2 shows a negative association with inflammatory disease [21, 24]. It was found that cultured macrophages from *Ptpn2*^−/−^ mice were hypersensitive to LPS and that decreased expression of PTPN2 enhanced the secretion of monocyte chemoattractant protein (MCP-1) and interleukin 6 (IL-6) [25, 26].

The role of PTPN2 in T2DM and its relationship to VDR have not been explored. Recently, the Diabetes Autoimmunity Study in the Young (DAISY) reported an interaction between a PTPN2 variant PTPN2 rs1893217 and a functional VDR variant VDR rs2228570 which is associated with progression of T1DM [27]. The anti-inflammatory role of VDR is widely recognized, but the exact mechanism of its action is not clear. Based on the findings that VDR interacts with PTPN2, we hypothesized that VDR may mediate anti-inflammatory effects by regulating PTPN2 which may be responsible for reducing inflammatory responses associated with diabetic kidney disease which in turn postpone the progression of diabetic kidney disease. We investigated the expression of PTPN2 in T2DM and its correlation with the severity of diabetic kidney disease, VDR, and inflammatory factors MCP-1, IL-6, and TNF*α*. We also verified the anti-inflammatory effects of PTPN2 in THP-1 cells in the presence of high glucose.

## 2. Materials and Methods

### 2.1. Recruitment of T2DM Patients and Healthy Controls

According to the World Health Organization (WHO) 1999 standard [28], we recruited 101 T2DM patients from the Departments of Nephrology and Endocrinology at the Third Xiangya Hospital, Central South University, China, from 2014 to 2015. Patients with the following conditions were excluded: T1DM, secondary diabetes, diabetic acute complications (such as diabetic ketoacidosis and hypertonic coma), estimated glomerular filtration rate (eGFR) < 60 mL/min/1.73 m^2^, infection, and severe cardiovascular and cerebrovascular diseases 3–6 months before recruitment. We also recruited 30 age- and gender-matched healthy adults as controls (NC). The clinical parameters of each study subject were collected and analyzed. Qualified T2DM patients were divided into three groups based on their spot urinary albumin-to-creatinine ratio (uACR): the normal albuminuria group (normo, uACR < 30 mg/g; *n* = 29), the microalbuminuria group (micro, 30 mg/g ≤ uACR < 300 mg/g; *n* = 34), and the macroalbuminuria group (macro, uACR ≥ 300 mg/g; *n* = 38). The study was carried out in accordance with the Declaration of Helsinki (2013) of the World Medical Association. Written informed consent was obtained from all study participants, and the study protocol was approved by the Ethics Committee of the Third Xiangya Hospital of Central South University (Changsha, China).

### 2.2. Sample Collection

Peripheral venous blood samples were collected from all 131 participants, including 101 T2DM patients and 30 healthy controls, after overnight fasting (at least 8 hours). PBMCs, including monocytes, lymphocytes, and other leukocytes, were isolated by Percoll continuous density gradient separation from the blood samples as previously described [29]. Serum biochemical indices were measured by automatic biochemical analyzers (Hitachi 7600). Spot morning urine samples were collected from the 101 T2DM patients, centrifuged, and stored at −20°C for further analyses. uACR was calculated as urinary albumin concentration divided by urinary creatinine concentration.

### 2.3. Cell Culture, Treatment, and Transfection

Human acute monocytic leukemia cells (THP-1) were purchased from the Cell Biology Department of Central South University. Cells were cultured in RPMI 1640 medium (Hyclone, New York, USA) supplemented with 10% fetal bovine serum (FBS), 100 *μ*g/mL streptomycin, and 100 U/mL penicillin (Life Technologies, New York, USA) at 37°C in a humidified atmosphere of 5% CO_2_ and 95% air.

Cells were seeded into 12-well culture plates for 24 h and then incubated with or without paricalcitol (0.2 ng/mL) for 6 h. Cells were further stimulated with high glucose (30 mmol/L) for 48 h. For transfection experiments, cells were seeded into 12-well culture plates in complete medium without penicillin or streptomycin for 24 h. Cells were transfected with either siRNA against PTPN2 or with scrambled control siRNA using Lipofectamine 2000 (Life Technologies, New York, USA) according to the manufacturer's instructions. Six hours after transfection, the culture medium was replaced with fresh medium and cells were divided into five groups: siCtrl group, siCtrl + HG group (high glucose 30 mmol/L for 48 h), siCtrl + HG + PTPN2 group (recombined human PTPN2 50 ng/mL for 24 h), HG + siPTPN2 group, and HG + siPTPN2 + pari group (paricalcitol 0.2 ng/mL).

### 2.4. Western Blot

Cells were lysed with buffer containing 20 mM Tris-HCl (pH 7.4), 4% sodium dodecyl sulfate, and 10% glycerol. Lysates were boiled at 100°C for 10 minutes. Protein concentration was determined with the BCA Protein Assay Kit (Pierce, USA). For Western blot analysis, protein samples were separated on a 10% SDS-PAGE gel and transferred to polyvinylidene difluoride membranes (Millipore, Bedford, USA). Membranes were incubated with specific primary antibodies against PTPN2 (Abcam, 1 : 1000, Cambridge, UK), VDR (Santa Cruz, 1 : 200, Dallas, USA), and GAPDH (Abcam, 1 : 10000, Cambridge, UK) overnight at 4°C. Horseradish peroxidase- (HRP-) labeled secondary antibodies were added for 1 h at room temperature. Signal was developed using ECL Plus Western Blotting Detection Reagents (Advansta, Menlo Park, CA, USA) and X-ray film (Kodak, Rochester, NY, USA). Bands were quantified using ImageJ software.

### 2.5. Real-Time PCR

Total RNA was isolated from THP-1 cells using Trizol (Thermo, New York, USA) reagent according to the manufacturer's instruction. First-strand cDNAs were synthesized from 2 *μ*g of total RNA in a 20 *μ*L reaction using the Revert Aid First Strand cDNA Synthesis Kit. Specific primers used were PTPN2 (forward: 5′-ATCGAGCGGGAGTTCGA-3′; reverse: 5′-TCTGGAAACTTGGCCACTC-3′), VDR (forward: 5′-AGTGCAGAGGAAGCGGGAGATG-3′; reverse: 5′-CTGGCAGAAGTCGGAGTAGGTG-3′), MCP-1 (forward: 5′-CTCAGCCAGATGCAATCAAT-3′; reverse: 5′-GCTTCTTTGGGACACTTGCT-3′), IL-6 (forward: 5′-CCCCTGACCCAACCACA-3′; reverse: 5′-TGCCGAAGAGCCCTCA-3′), TNF*α* (forward: 5′-AGCTCCAGTGGCTGAACCG-3′; reverse: 5′-TGGTAGGAGACGGCGATGC-3′), and GAPDH (forward: 5′-CAGCCTCAAGATCATCAGCAA-3′; reverse: 5′-TGTGGTCATGAGTCCTTCCA-3′) and were designed based on the gene sequences and synthesized by Generay Biotech. Real-time reverse transcription- (RT-) PCR quantification for individual target mRNA expression was performed with the CFX96 Real-Time Detection System (Bio-Rad, Hercules, CA, USA) using a Takara SYBR green real-time PCR kit (Takara, Japan). The amount of specific mRNA in each sample was calculated from the standard curve and normalized GAPDH mRNA. The comparative 2^−ΔΔCT^ method was used for quantification and statistical analysis.

### 2.6. ELISA

Serum levels of PTPN2 and IL-6 in T2DM patients and levels of MCP-1, IL-6, and TNF*α* in cell culture supernatant were determined with ELISA kits (R&D, Minnesota, USA).

### 2.7. Statistical Analysis

All data were analyzed using SPSS 19.0 statistical software and presented as mean ± SD (standard derivation). Difference between the two groups was tested using *t*-test. Differences among 3 or more groups were tested by one-way ANOVA. Spearman correlation and stepwise multiple linear regression analyses were used to determine the correlations between PTPN2 and uACR, VDR, or other variables. In particular, because the value of uACR is nonnormally distributed, its value was used for performing Ln transformation in correlation analysis. *P* < 0.05 was considered statistically significant.

## 3. Results

### 3.1. Clinical and Biochemical Data of the Study Participants

To investigate the correlation between PTPN2 in PBMCs and the severity of albuminuria, we minimized the variables in the study subjects by selecting patients and healthy individuals with comparable parameters (Table 1). There were no statistical differences in age, gender, BMI, hemoglobin, or calcium levels among all the four groups. Moreover, no difference in the duration of disease, serum albumin (ALB), HbA1c, and total cholesterol (TC) was observed in the three T2DM groups. However, compared to the NC group, the T2DM groups had dramatically higher levels of fasting blood glucose (FBG), eGFR, uACR, and IL-6 and a lower level of serum albumin (ALB) and VDR mRNA. We observed increased triglyceride (TG), eGFR, uACR, and IL-6 and decreased 25(OH)D, and VDR mRNA in the macro group was particularly prominent.

### 3.2. PTPN2 Is Downregulated in Serum and PBMCs Isolated from T2DM Patients and Is Inversely Correlated with the Severity of Albuminuria

**Figure 1:**
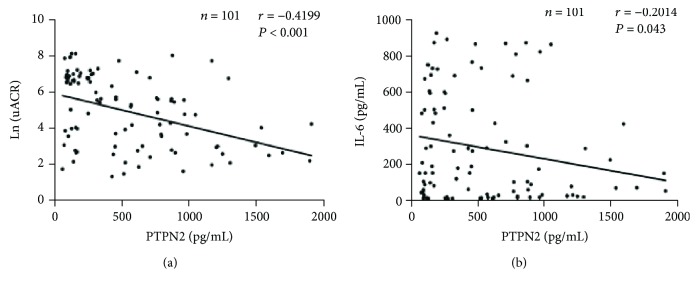
PTPN2 expression in serum is downregulated in T2DM and inversely correlated with uACR and IL-6. (a) PTPN2 protein levels in NC (*n* = 30), normo (*n* = 29), micro (*n* = 34), and macro (*n* = 38) groups were quantified by ELISA. Scatterplot shows an inverse relationship between PTPN2 protein levels and uACR (*n* = 101; *r* = −0.4199; *P* < 0.001). (b) Scatterplot showing an inverse relationship between PTPN2 and IL-6 in protein levels (*n* = 101; *r* = −0.2014; *P* = 0.043). Data represents mean ± SD.

We first analyzed the PTPN2 level in the serum of all subjects. Expression levels of PTPN2 decreased with an increase in uACR (Figure 1(a)). We next used Spearman correlation analysis to study the relationship between serum PTPN2 levels and uACR in T2DM patients, an indicator for the severity of albuminuria. As shown in Figure 1(a), serum PTPN2 protein (*n* = 101, including all the three diabetic groups) levels were inversely correlated with uACR in these patients (*r* = −0.4199, *P* < 0.001). After adjusting the potential confounding factors (FBG, SBP, DBP, ALB, TG, TC, 25(OH)D, eGFR, and IL-6), multiple stepwise regression analysis showed that PTPN2 protein (*β* = −0.398, *P* < 0.001) remained inversely associated with the uACR levels, an indication of increased risk of kidney malfunction. PTPN2 mRNA showed a positive correlation with both VDR mRNA (*β* = 0.577, *P* = 0.022) and 25(OH)D (*β* = 0.185, *P* < 0.001). Diabetic kidney disease is a microvascular disease with low-grade chronic inflammation [30]. We showed that the level of IL-6 was significantly higher in T2DM patients than in the healthy controls, and Spearman correlation analysis revealed that PTPN2 negatively correlated with IL-6 (*r* = −0.2014, *P* = 0.043) (Figure 1(b)).

**Figure 2:**
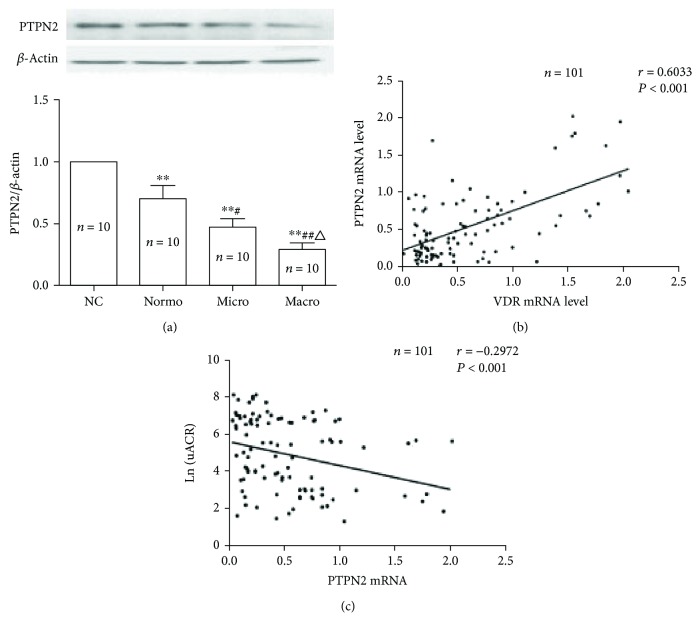
PTPN2 expression in PBMCs is downregulated in T2DM and positively correlated with VDR. (a) PTPN2 protein levels determined by Western blot (*n* = 10 in each group, age- and gender-matched); *β*-actin was used as loading controls. (b) PTPN2 mRNA levels in NC (*n* = 30), normo (*n* = 29), micro (*n* = 34), and macro (*n* = 38) groups were quantified by real-time RT-qPCR. Scatterplot showing a positive relationship between PTPN2 mRNA levels and VDR mRNA level (*n* = 101; *r* = 0.6033; *P* < 0.001). (c) Scatterplot showing inverse relationship between PTPN2 mRNA levels and uACR level (*n* = 101; *r* = −0.2972; *P* < 0.001). Data represents mean ± SD versus NC (^∗∗^*P* < 0.01), versus normo (^#^*P* < 0.05, ^##^*P* < 0.01), and versus micro (^△^*P* < 0.05).

To analyze the correlation between PTPN2 and VDR, we measured PTPN2 and VDR mRNA levels in PBMCs isolated from all subjects. Protein samples from ten subjects from each group were randomly selected for Western blotting. Both protein and mRNA levels of PTPN2 and VDR in PBMCs derived from the normo, micro, and macro groups were significantly lower when compared to the NC group (Figures 2(a) and 2(b)). Among the three diabetic groups, the normo group had the highest levels of both PTPN2 and VDR, whereas the macro group had the lowest levels (Figures 2(a) and 2(b)). Spearman correlation analysis showed that PTPN2 mRNA (*n* = 101) levels were positively correlated with VDR mRNA levels (*r* = 0.6033, *P*<0.001) (Figure 2(b)) but inversely correlated with uACR (*r* = −0.2972, *P*<0.001) (Figure 2(c)) in these patients. Taken together, these results indicate that reduced PTPN2 expression is independently associated with the degree of albuminuria and VDR level in T2DM patients.

### 3.3. High Glucose Increased Inflammatory Factors and Decreased PTPN2 Expression and These Changes Were Reversed by VDR Induction

**Figure 3:**
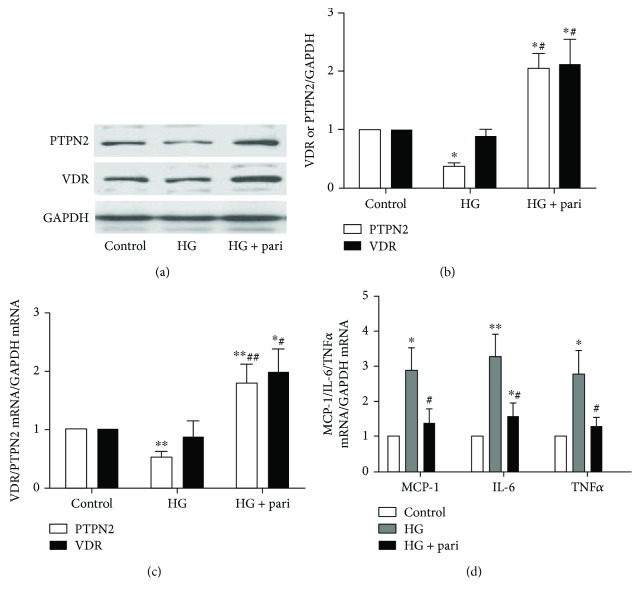
High glucose downregulated PTPN2 in THP-1 cells, and this is reversed by increasing VDR level. THP-1 cells were stimulated with high glucose (30 mmol/L) for 48 hours (HG). Cells were pretreated with paricalcitol (0.2 ng/mL) (HG + pari) for 6 hours before HG. (a, b) PTPN2 and VDR protein levels as determined by Western blot; GAPDH was used as a loading control. (c) PTPN2 and VDR mRNA levels as quantified by real-time RT-qPCR. (d) ELISA for inflammatory cytokines MCP-1, IL-6, and TNF*α*. Experiments were repeated three times, and data represent mean ± SD versus control (^∗^*P* < 0.05, ^∗∗^*P* < 0.01) and versus HG (^#^*P* < 0.05, ^##^*P* < 0.01).

Cultured THP-1 cells stimulated with glucose produced a significant inflammatory response (Figure 3(d)) which was accompanied by a decreased PTPN2 at both protein and mRNA levels. But VDR expression did not change significantly (Figures 3(a) and 3(b)). Under the stimulation of high glucose, intervention with paricalcitol significantly upregulated VDR and PTPN2 expression (Figures 3(a) and 3(b)) and reduced the level of inflammatory cytokines (Figure 3(d)). These data indicate that inflammation can change the expression of PTPN2 and its expression is regulated by VDR.

### 3.4. PTPN2 Has Anti-Inflammatory Activities and the Anti-Inflammatory Activity of VDR Is Partially Dependent on PTPN2

**Figure 4:**
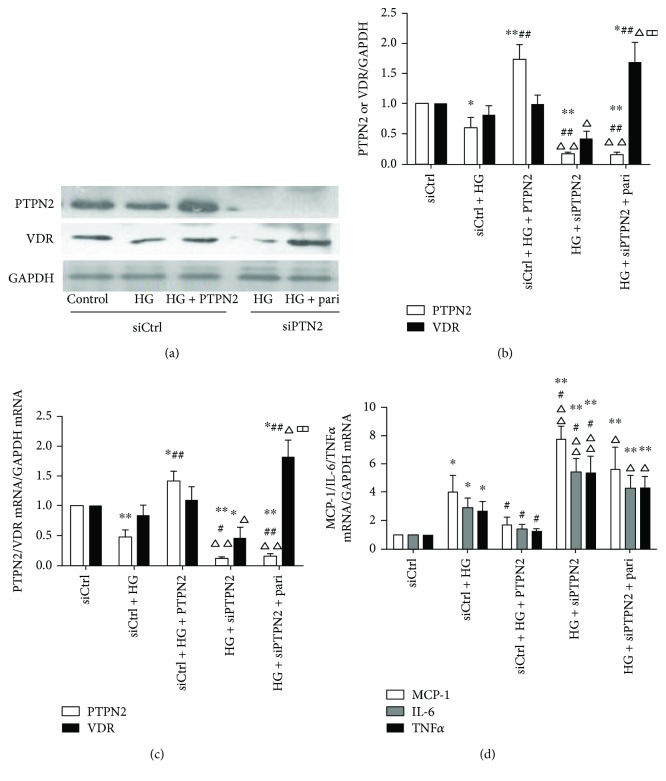
PTPN2 inhibits inflammatory factors induced by high glucose in THP-1 cells. siCtrl and siPTPN2 THP-1 cells were stimulated with high glucose (30 mmol/L) for 48 hours and then treated with or without paricalcitol (0.2 ng/mL for 6 hours) or recombined human PTPN2 (50 ng/mL for 24 hours). (a, b) PTPN2 and VDR protein levels were determined by Western blot; GAPDH was used as a loading control. (c) PTPN2 and VDR mRNA levels were quantified by real-time RT-PCR. (d) Real-time RT-PCR of inflammatory cytokines MCP-1, IL-6, and TNF*α*. Experiments were repeated three times, and data represent mean ± SD versus siCtrl (^∗^*P* < 0.05, ^∗∗^*P* < 0.01), versus siCtrl + HG (^#^*P* < 0.05, ^##^*P* < 0.01), versus siCtrl + HG + PTPN2 (^△^*P* < 0.05, ^△△^*P* < 0.01), and versus HG + siPTPN2 (^□□^*P* < 0.01).

Knockdown of PTPN2 (Figures 4(a)–4(c)) further elevated the protein and mRNA levels of inflammatory cytokines by high-glucose treatment in THP-1 cells (Figure 4(d) and Table 2). Moreover, paricalcitol failed to exert its anti-inflammatory effect when PTPN2 was knocked down. Next, we treated the cells with recombined human PTPN2 to upregulate its expression. We found that PTPN2 suppressed the expression of MCP-1, IL6, and TNF*α* mediated by HG. All these results demonstrated that PTPN2 has anti-inflammatory activities and the anti-inflammatory utility of VDR is partially dependent on PTPN2.

## 4. Discussion

In this report, we investigated the relationship between VDR and PTPN2 expression and between PTPN2 and the severity of albuminuria and inflammation in T2DM. First, we showed that PTPN2 expression in serum and PBMCs was much lower in T2DM patients than in healthy adults. In contrast, elevated serum levels of IL-6 were observed in T2DM. PTPN2 was negatively correlated with uACR and IL-6. Our previous studies showed that VDR expression in renal biopsy tissues and PBMCs was significantly downregulated in T2DM patients [31]. Our current results showed a similar profile for PTPN2 in T2DM patients and that PTPN2 was positively correlated with VDR. Multiple stepwise regression analysis and correlation analysis demonstrated that a reduction of PTPN2 is associated with lower VDR and higher uACR, a major indicator for assessing the development of diabetic kidney disease. Our study has limitations. Due to the difficulty in obtaining renal biopsies from T2DM patients, we could not demonstrate the expression of PTPN2 in renal tissues or analyze the correlation between PTPN2 expression in renal tissues and the severity of albuminuria and inflammation. Moreover, the correlation between PTPN2 in PBMCs and the severity of albuminuria was calculated in a cross-sectional study. Also, the number of PBMCs used in this study was small. Thus, future prospective longitudinal studies focused on larger sample quantity are needed to further confirm these observations.

The role of inflammation in the pathogenesis of T2DM and its associated complications is now well established [30, 32, 33]. PTPN2 is involved in T1DM, modulates pancreatic *β*-cell apoptosis [34], controls CD4^+^ T cell differentiation, and limits intestinal inflammation [35]. At present, there are many reports of PTPN2-knockout mouse model to study diabetes. The phenotype of PTPN2-knockout mice varies according to its background. PTPN2-knockout C57BL/6 mice have a normal lifespan but showed a reduction in obesity symptoms and increased insulin sensitivity [36], but PTPN2-knockout BALB/C mice exhibited a significant systemic inflammatory response, and a large amount of IL-12, IFN*γ*, and TNF*α* infiltrated in the spleen and nonlymphoid tissues [25, 37]. Pancreas-specific-PTPN2-knockout mice exhibited impaired glucose tolerance during normal dietary feeding and remarkable impaired glucose tolerance and decreased insulin secretion during high-fat diets [38]. It was reported that deficiency of PTPN2 in the pancreas aggravated apoptosis induced by IL-1*β* and IFN*γ* and also promoted IFN*γ*-induced phosphorylation of STAT1-inducing *β*-cell death [39]. These findings suggest that PTPN2 is an important regulator of diabetes and inflammation. This is the first report, to our knowledge, linking PTPN2 expression levels to inflammation in T2DM. Here, we showed a significant decrease in PTPN2 expression in serum and PBMCs from T2DM patients, with the lowest level seen in the macro group. TNF*α*, IL-6, and MCP-1 are important inflammatory mediators that are upregulated in T2DM patients [30]. Our results are in agreement with these observations. Furthermore, we showed that PTPN2 is inversely proportional to IL-6 but positively associated with anti-inflammatory VDR. Our *in vitro* experiments demonstrated that exogenous PTPN2 downregulated these inflammatory markers. Deficiency of PTPN2 further aggravated inflammation induced by high glucose. This confirms the anti-inflammatory properties of PTPN2. Taken together, our data showed that high blood glucose can downregulate PTPN2 and inflammation may play an important role in it. In contrast, several *in vivo* studies show that the expression of PTPN2 increased in epithelial cells like HK2 with the stimulation of high glucose [21, 40, 41]. These discrepancies may be due to difference in cell types that express PTPN2 and how they regulate inflammation. Constitutive expression of PTPN2 was stronger in THP-1 monocytes than in other epithelial cells, so it may be consumed at first and then increase synthesis after an ongoing stimulation. Of course, this bold hypothesis needs to be confirmed by more experiments.

Vitamin D-VDR signaling is associated with a strong anti-inflammatory activity. Activated VDR can reduce the expression of TNF*α* by inhibiting p65 nuclear translocation and NF-*κ*B activation [42]. The Diabetes Autoimmunity Study in the Young (DAISY) reported an interaction between sequence variants at PTPN2 and VDR as being associated with the risk of T1DM progression in children with islet autoantibodies [27]. Such an interaction is mechanistically consistent with the presence of vitamin D-responsive elements (VDREs) across the PTPN2 locus and the observation that PTPN2 expression in lymphoblastoid cell lines is upregulated when exposed to the VDR ligand calcitriol [43]. Our study showed that anti-inflammatory effects of VDR were suppressed in PTPN2-deficient cells. Therefore, it is conceivable that when the levels of inflammatory factors are elevated in T2DM, PTPN2 expression is downregulated in immunocytes but upregulated in epithelial cells (as seen in other studies) and acts synergistically with VDR to attenuate the inflammation and protect against renal injury. Further studies are needed to test this hypothesis.

The mechanism of interaction between PTPN2 and VDR in T2DM is not entirely clear. Future studies should be carried out to test the anti-inflammatory effects of PTPN2 on T2DM in the laboratory. This strategy is both promising and challenging.

## Figures and Tables

**Table 1:** Clinical parameters ($\bar{X}\pm S$) of study participants.

Subjects	NC	Normo	Micro	Macro
*n*	30	29	34	38
Gender, M/F	14/16	16/13	19/15	25/13
Age, y	52.34 ± 16.82	53.92 ± 11.92	51.82 ± 13.53	52.42 ± 18.13
BMI, kg/m^2^	23.01 ± 3.92	23.73 ± 4.02	24.52 ± 3.89	23.27 ± 3.15
Duration of disease, y	—	6.92 ± 5.79	7.39 ± 6.28	8.11 ± 6.23
SBP, mmHg	123.3 ± 11.7	126.8 ± 12.3	132.8 ± 16.8^∗^	148.6 ± 19.2^∗^^#^
DBP, mmHg	72.8 ± 9.7	73.8 ± 11.9	84.2 ± 10.4^∗^^#^	85.9 ± 13.3^∗^^#^
ALB, g/L	46.68 ± 4.98	40.23 ± 5.18^∗^	39.43 ± 6.92^∗^	38.23 ± 7.90^∗^
Hb, g/L	135.8 ± 15.9	138.5 ± 14.8	136.6 ± 15.2	127.3 ± 21.6
TG, mmol/L	2.11 ± 2.09	2.03 ± 1.83	2.21 ± 2.05	2.98 ± 2.82^∗^^#△^
TC, mmol/L	4.37 ± 1.03	4.73 ± 2.85	4.88 ± 2.05	5.07 ± 2.85^∗^
FBG, mmol/L	5.03 ± 1.64	8.01 ± 2.66^∗^	8.49 ± 2.38^∗^	8.20 ± 2.55^∗^
HbA1c, %	—	8.49 ± 2.03	8.99 ± 2.58	9.23 ± 1.98
Calcium, mmol/L	2.33 ± 0.19	2.12 ± 0.18	2.11 ± 0.14	2.29 ± 0.12
25(OH)D, ng/mL	21.72 ± 3.92	19.63 ± 3.72	18.76 ± 7.92	14.20 ± 6.86^∗^^#△^
IL-6, pg/mL	47.82 ± 39.77	136.50 ± 129.60^∗^	327.39 ± 318.36^∗∗^^#^	370.39 ± 328.53^∗∗^^#^
eGFR, mL/min	112.24 ± 12.84	118.63 ± 29.83	113.53 ± 31.84	82.63 ± 23.62^∗^^#△^
uACR, *μ*g/mg	8.98 ± 3.62	13.80 ± 7.35^∗^	138.52 ± 96.31^∗^^#^	1263.11 ± 787.77^∗∗^^##△△^
VDR mRNA in PBMC	1.24 ± 0.62	0.82 ± 0.52^∗^	0.62 ± 0.56^∗^^#^	0.39 ± 0.36^∗^^#△^

M: male; F: female; —: no data. Results are expressed as mean ± SD or ratio. Compared with the NC group, ^∗^*P* < 0.05, ^∗∗^*P* < 0.01; compared with the normo group, ^#^*P* < 0.05, ^##^*P* < 0.01; compared with the micro group, ^Δ^*P* < 0 .05, ^ΔΔ^*P* < 0.01.

**Table 2:** ELISA of inflammatory cytokines stimulated with high glucose after PTPN2 silencing.

Subjects	siCtrl	siCtrl + HG	siCtrl + HG + PTPN2	HG + siPTPN2	HG + siPTPN2 + pari
MCP-1 (pg/mL)	91.5 ± 39.1	252.9 ± 96.9^∗∗^	148.3 ± 80.6^#^	403.8 ± 168.7^∗∗^^#ΔΔ^	369.2 ± 87.5^∗^^ΔΔ^
IL-6 (pg/mL)	3.26 ± 1.58	15.9 ± 4.86^∗∗^	9.04 ± 4.99^∗^^#^	25.9 ± 9.15^∗∗^^##ΔΔ^	24.6 ± 11.4^∗∗^^ΔΔ^
TNF*α* (pg/mL)	7.66 ± 4.32	26.3 ± 11.5^∗∗^	14.2 ± 4.22^∗^^#^	43.7 ± 18.9^∗∗^^#ΔΔ^	35.0 ± 20.2^∗∗^^ΔΔ^

Results are expressed as mean ± SD or ratio. Compared with the siCtrl group: ^∗^*P* < 0.05, ^∗∗^*P* < 0.01; compared with the siCtrl + HG group: ^#^*P* < 0.05, ^##^*P* < 0.01; compared with the siCtrl + HG + PTPN2 group: ^ΔΔ^*P* < 0.01.
